# Effect of introducing a pharmacist-led support system on the administration rate of vancomycin loading dose and the 0–24-hour area under the concentration–time curve

**DOI:** 10.1017/ash.2025.10181

**Published:** 2025-10-14

**Authors:** Tatsuya Tai, Takahiro Motoki, Masahiro Watanabe, Naohiro Kurokawa, Sayaka Yamashita, Kazunori Yamaguchi, Hiroaki Tanaka, Yuichi Muraki, Shinji Kosaka

**Affiliations:** 1 Department of Infection Control Service Office, Kagawa University Hospital, Kagawa, Japan; 2 Department of Pharmacy, Kagawa University Hospital, Kagawa, Japan; 3 Department of Safety Management, Kagawa University Hospital, Kagawa, Japan; 4 Department of Pharmacology, School of Pharmacy, Shujitsu University, Okayama, Japan; 5 Laboratory of Clinical Pharmacoepidemiology, Kyoto Pharmaceutical University, Kyoto, Japan

## Abstract

**Objective:**

This study aimed to evaluate the impact of a pharmacist-led support system on the administration rate of vancomycin (VCM) loading dose, 0–24-hour area under the concentration–time curve (AUC_0–24_), incidence of acute kidney injury (AKI), and all-cause mortality in hospitalized patients.

**Design:**

This retrospective study with interrupted time series analysis was conducted from January 2021 to May 2024.

**Setting:**

A public tertiary referral center providing acute and specialized inpatient care in Japan.

**Patients:**

Among the 587 hospitalized patients who received VCM during the study period, 326 were evaluated.

**Intervention:**

The intervention comprised implementation of a pharmacist-led support system involving prospective prescription review and direct intervention when a VCM loading dose (25–30 mg/kg) was not prescribed.

**Results:**

The loading dose administration rate increased significantly by 43.2% immediately introducing the support system (95% confidence interval: 8.40–77.90; *P* = 0.0156), without significant trend change thereafter. AUC_0–24_ also increased significantly (241.0 vs 307.0; *P* < 0.001); there were no significant differences in AKI incidence or 90-day mortality.

**Conclusions:**

The support system improved the loading dose administration rate and AUC_0–24_ without significantly increasing AKI or mortality. The improvement was immediate and sustained over 122 weeks, supporting its use in institutions aiming to optimize VCM loading doses where evidence remains limited.

## Introduction

Vancomycin (VCM) is commonly used for treating gram-positive bacterial infections, including methicillin-resistant *Staphylococcus aureus*. High doses of VCM have been associated with acute kidney injury (AKI), primarily through mechanisms involving oxidative stress and mitochondrial damage.^1^ Dosing strategies based on the area under the concentration–time curve (AUC) and trough serum concentrations (Cmin) have been developed to help balance efficacy and toxicity.^2,3^

Previous therapeutic drug monitoring guidelines used Cmin as a surrogate marker for AUC;^4^ however, recent studies have highlighted discrepancies between these 2 parameters.^5–7^ As a result, AUC-based dosing has been recommended in the updated guidelines released in the United States in May 2020 and in Japan in February 2022.^8–10^

Evidence suggests that appropriate loading doses do not increase the risk of renal impairment and can improve treatment success, particularly in patients with an estimated glomerular filtration rate of 30–80 mL/min/1.73m^2^.^11^ Nevertheless, the administration rate of the loading dose remains suboptimal in clinical practice, limiting the achievement of target AUC levels in the early phase of therapy.^12^ For instance, a loading dose was implemented in less than one-third of cases in an Australian public hospital.^13^ Furthermore, underdosing was reported in approximately 70% of cases at a Belgian tertiary care center.^14^

Pharmacist-led interventions have recently gained attention as a strategy for supporting appropriate antimicrobial use by assisting physicians in designing a VCM dosing support system.^15,16^ However, there is limited evidence on the impact of such support on clinical outcomes, including loading dose administration, AUC_0–24_, AKI incidence, and mortality. This study aimed to evaluate the effects of a pharmacist-led support system for initial VCM dosing introduced at Kagawa University Hospital, focusing on its impact on loading dose administration, AUC_0–24_, AKI incidence, and all-cause mortality.

## Methods

### Facility overview and description of the support system

At our institution, VCM serum concentrations are measured 24/7 using the ARCHITECT i2000 SR immunoassay system. Pharmacists estimate Cmin and AUC values using EasyTDM Ver. 3-6-1-1 M (Kagawa Society of Hospital Pharmacists, Japan).^17^ Ward pharmacists performed blood concentration evaluations on weekdays; on-duty pharmacists during nights and holidays.

In line with the 2022 guideline revision, the institution transitioned from Cmin-based to AUC-based dosing. These changes, implemented in February 2022, included pharmacist intervention when a loading dose was not prescribed or provided, with direct feedback to the prescribing physician. The Antimicrobial Stewardship Team pharmacists also reviewed prescriptions from the day of ordering to the next business day. Pharmacists, including but not limited to Antimicrobial Stewardship Team members, provided AUC-based dosing recommendations—covering both loading and maintenance doses—based on pharmacokinetic calculations or the renal function–adjusted dosing nomogram described in the Japanese Society of Therapeutic Drug Monitoring guidelines. ^9^ Pharmacists involved in dosing receive regular training; to support consistent administration, system updates are shared annually with the entire hospital staff.

### Study population and study period

This retrospective study included patients who received VCM over 179 weeks from January 1, 2021, to May 31, 2024. A total of 587 cases of intravenous VCM prescription were identified during the study period. Of these, 117, 58, and 18 cases were excluded due to discontinuing therapy was within 2 days of initiation, ongoing renal replacement therapy (hemodialysis or peritoneal dialysis), and presence of AKI prior to VCM initiation, respectively. In addition, 32, 4, and 32 cases were excluded because they were younger than 15 years, VCM was initiated at another facility, and administration was not via the intravenous route, respectively. After these exclusions, 326 patients met the inclusion criteria and were evaluated. The study period was divided into a preintervention phase (57 wk: January 1, 2021–February 3, 2022) and a postintervention phase (122 wk: February 4, 2022–May 31, 2024), considering the date of introducing the system. The exclusion criteria included patients who completed VCM within 2 days after initiation; younger than 15 years; on continuous renal replacement therapy, hemodialysis, or continuous ambulatory peritoneal dialysis; who did not receive intravenous administration; with AKI before VCM administration; and whose VCM was initiated at another facility.

### Comparison of patient background characteristics before and after introducing the support system

We compared the background characteristics of patients receiving VCM before and after the introduction of the support system. The following parameters were evaluated on the day of initiating VCM therapy: age, sex, body weight, serum creatinine, body mass index, blood urea nitrogen/creatinine ratio, disease severity assessed by the Pitt Bacteremia Score, distribution of causative microorganisms (including methicillin-resistant *Staphylococcus aureus*), and the concomitant use of nephrotoxic agents. The concomitant use of nephrotoxic agents—defined as the use of any of the following: diuretics, non-steroidal anti-inflammatory drugs, aminoglycoside antibiotics, amphotericin B, or tazobactam/piperacillin—was evaluated as a single composite variable.

### Impact of the support system on loading dose administration rate

To assess the system’s impact, the biweekly administration rate of the loading dose was evaluated using a quasi-experimental interrupted time series analysis. The loading dose was defined as an initial dose of 25–30 mg/kg.

### Impact of the support system on AUC optimization

We compared the impact of the new system on the optimization of VCM AUC before and after its introduction. AUCs were evaluated using AUC_0–24_, AUC_24–48_ (24–48-h AUC), and AUC_ss (steady-state AUC), all of which were calculated based on measured serum concentrations. Individual VCM AUCs were calculated by simulated concentration–time profiles generated by Bayesian estimation with EasyTDM. AUCs were determined using the trapezoidal rule, considering a 2-compartment model and each pharmacokinetic phase, including drug administration, distribution, and elimination.^18^

### Impact of the support system on patient outcomes

The impact of the system on patient outcomes was assessed by comparing data before and after its implementation. Outcomes included the incidence of adverse events such as AKI and 90-day all-cause mortality. AKI was defined as an increase in serum creatinine of ≥0.3 mg/dL or ≥50% from baseline in 2 consecutive measurements.^18^ Other adverse events, including liver dysfunction, leukopenia, and thrombocytopenia, were evaluated based on the criteria of the Japanese Society of Chemotherapy Antimicrobial Agents Safety Evaluation Committee.^19^

### Statistical analysis

The effects of the system on AUC and patient outcomes were evaluated using Fisher’s exact test for categorical variables and the Mann–Whitney *U* test for continuous variables. A *P* value of <.05 was considered statistically significant. A quasi-experimental interrupted time series analysis was conducted using a seasonal autoregressive integrated moving average model to adjust for baseline trends, autocorrelation, seasonality, and changes in level and slope before and after introducing the pharmacist-led support system.^20,21^ Statistical analyses were performed using R software (version 4.4.3; R Foundation for Statistical Computing, Vienna, Austria), with the seasonal autoregressive integrated moving average model implemented via the Forecast package.

### Ethics statements

Informed consent was obtained via an opt-out on the website in accordance with the Ethical Guidelines for Medical and Biological Research Involving Human Subjects in Japan. This study was conducted in compliance with the “Clinical Guidelines for Medical Research Involving Human Subjects” and approved by the Ethics Committee (Clinical Research Review Committee) established at our hospital (approval number 2024-175).

## Results

### Comparison of patient background characteristics before and after introducing the support system

Among the 587 patients who received VCM, 326 met the inclusion criteria and were evaluated. There were no significant differences in patient background characteristics or causative organisms between the pre- and postintervention periods (Table 1). Methicillin-resistant *Staphylococcus aureus* was identified in 26.6% and 21.2% of the patients in the preimplementation and postimplementation groups, respectively.

**Table 1.** Comparison of patient background characteristics before and after introducing the support system

	Presupport system group (n=109)	Postsupport system group (n=217)	*P* value
Age (years old) ^a)^	69.0 (59.0–77.0)	72.0 (60.0–79.0)	.261
Sex (male)	66 (60.6%)	137 (63.1%)	.717
Body weight (kg) ^a)^	53.0 (43.2–62.9)	54.5 (47.2–63.0)	.142
Scr (mg/dl) ^a)^	.7 (.5–.9)	.7 (.5–.9)	.353
BMI (kg/m^2^) ^a)^	20.6 (17.1–23.7)	21.3 (18.5–24.3)	.088
BUN/Scr ratio	25.3 (18.5–34.3)	23.6 (17.1–34.9)	.399
PBS ^a)^	.0 (.0–2.0)	1.0 (.0–2.0)	.531
The concomitant use of nephrotoxic agents	77 (70.6%)	134 (61.6 5)	.140
Causative microorganisms			
MRSA	29 (26.6%)	46 (21.2%)	.329
MSSA	2 (1.8%)	6 (2.8%)	.723
*Staphylococcus lugdunensis*	1 (.9%)	2 (.9%)	1.000
*Corynebacterium* sp.	12 (11.0%)	17 (7.8%)	.410
*Bacillus cereus*	6 (5.5%)	17 (7.8%)	.500
Coagulase-negative *Staphylococci*	17 (15.6%)	53 (24.4%)	.086
*Enterococcus faecium*	20 (18.3%)	28 (12.9%)	.190
Other	0 (0%)	2 (.9%)	.554
None	22 (20.2%)	46 (21.2%)	.886

For the percentage of each group for each item, a) median (25% IQR–75% IQR). Scr, serum creatinine; BMI, body mass index; BUN/Scr ratio, blood urea nitrogen/creatinine ratio; PBS, Pitt Bacteremia Score; MRSA, methicillin-resistant *Staphylococcus aureus*; MSSA, methicillin-sensitive *Staphylococcus aureus*.

### Impact of the support system on loading dose administration rate

**Figure 1.**
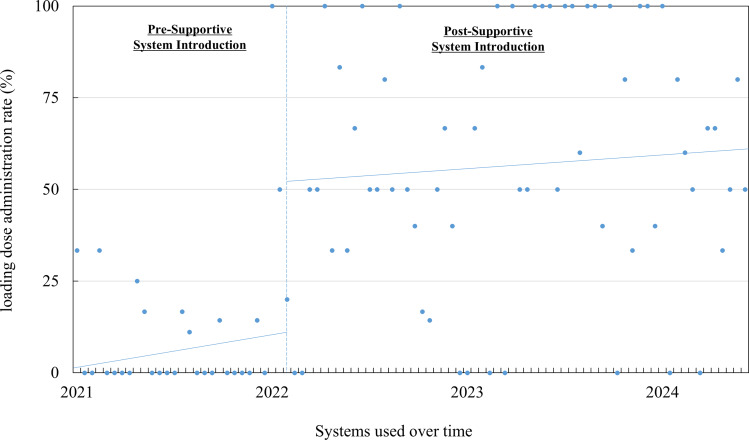
Time trend in loading dose administration rate following implementation of the support system.

Results from the quasi-experimental interrupted time series analysis are presented in Figure 1 and Table 2. During the preintervention period, the loading dose administration rate remained stable (0.32% per 2 wk; *P* = .342). After introducing the support system, an immediate and significant increase of 43.15% was observed (95% confidence interval: 8.40–77.90; *P* = .0156). No significant upward trend was observed during the postintervention period ( −.21% per 2 wk, 95% confidence interval: −0.9 to 0.5; *P* = .546).

**Table 2.** Impact of the support system on loading dose administration rate

	Baseline trend		Immediate change		Slope change	
	(95% CI)	*P* value	(95% CI)	*P* value	(95% CI)	*P* value
Loading dose administration rate	.32 (−.30 to 1.00)	.342	43.15 (8.40–77.90)	.016*	−.21 (−.90 to .50)	.546

CI, confidence interval.

### Influence of the support system on AUC optimization

Following the administration of the support system, both AUC_0–24_ and AUC_24–48_ values were significantly higher compared with the preintervention group (241.0 vs 307.0, *P* < .001; 424.0 vs 485.0, *P* = .018). However, there was no significant difference in AUC_ss between the groups (459.0 vs 486.0, *P* = .644) (Table 3).

**Table 3.** Impact of the support system on patient outcomes

Index	Presupport system group (n=109)	Postsupport system group (n=217)	Odds ratio	*P* value
AUC_0–24_^a)^	241.0 (203.0–299.0)	307.0 (231.0–367.0)	N/A	<.001^* b)^
AUC_24–48_^a)^	424.0 (336.0–559.0)	485.0 (389.0–577.0)	N/A	.018^* b)^
AUCss ^a)^	459.0 (372.0–618.0)	486.0 (379.0–594.0)	N/A	.644 ^b)^
90-d mortality	11 (10.1%)	17 (7.8%)	.7 [.3–1.9]	.532 ^c)^
AKI	14 (12.8%)	27 (12.4%)	1.0 [.5–2.1]	1.000 ^c)^
Thrombocytopenia	3 (2.8%)	2 (.9%)	.3 [.0–2.9]	.339 ^c)^
Leukopenia	2 (1.8%)	4 (1.8%)	1.0 [.1–11.3]	1.000 ^c)^
Liver dysfunction	7 (6.4%)	19 (8.8%)	1.4 [.5–4.1]	.523 ^c)^
No adverse events	87 (79.8%)	163 (75.1%)	1.3 [.7–2.4]	.405 ^c)^

For the percentage of each group for each item, a) median (25% IQR–75% IQR), b) Mann–Whitney *U* test, c) Fisher’s exact test. AUC, daily area under the concentration–time curve; AUC_0–24_, 0–24-hour AUC; AUC_24–48_, 24–48-hour AUC; AUCss, steady-state AUC; AKI, acute kidney injury; IQR, interquartile range. Some patients experienced multiple adverse events.

### Impact of the support system on patient outcomes

The 90-day mortality rate was 10.1% before and 7.8% after introducing the support system (*P* = .532), while the incidence of AKI was 12.8% and 12.4%, respectively (*P* = 1.000). No statistically significant differences were observed (Table 3).

## Discussion

This study uniquely assessed the impact of a newly implemented dosing system using quasi-experimental interrupted time series analysis. A significant and immediate increase in the loading dose rate was observed following administration, with the effect sustained over 122 weeks. The loading dose was associated with a significant improvement in AUC_0–24_, without increasing the incidence of AKI or mortality. These findings suggest that the system may safely and sustainably enhance the quality of initial VCM dosing through AUC optimization.

Previous studies have shown that a uniform loading dose, such as 1.75 g for *Staphylococcus aureus* bacteremia, can improve composite outcomes, including mortality, without increasing nephrotoxicity.^22^ Another report from an intensive care unit setting demonstrated that implementing a uniform 2 g loading dose increased the administration rate from 0% to 52.4% at 5 months and 63.9% at 16 months.^23^ However, uniform dosing may lead to overdosing and an increased risk of nephrotoxicity, particularly when Cmin exceeds 20 mg/L if renal function and body weight are not considered.^24^ In this study, we introduced a pharmacist-led support system that recommends an individualized loading dose instead of a fixed-dose approach. The implementation of the pharmacist-led support system led to an appropriate increase in loading dose administration, which remained stable at approximately 50%–60%. This rate is comparable to that reported in facilities using uniform dosing,^23^ suggesting that the support system has been established as standard practice and promotes sustained administration. However, because the system considers the physician’s final decision, further increases in the administration rate may have been limited.

These findings show that the appropriate increase in loading dose administration after introducing the support system significantly raised AUC_0–24_. Both groups achieved median AUC_(24–48)_ values ≥400 µg·h/mL, the efficacy threshold, suggesting that the postintervention group might have reached effective exposure earlier. From a safety perspective, renal toxicity has been associated with AUC_(24–48)_ ≥ 515 µg·h/mL and AUC_ss ≥ 600 µg·h/mL. Therefore, the recommended target AUC range is 400–500 µg·h/mL.^25^ In this study, although AUC_(24–48)_ increased significantly after administration, it remained within the target range. Moreover, AUC_ss did not change significantly. These findings align with previous reports indicating that loading doses do not affect AUC_ss,^26^ suggesting that the implemented support system improved efficacy while maintaining safety.

In this study, although a trend toward lower 90-day mortality was observed in the postintervention group, the difference was not statistically significant. Similarly, no significant difference in AKI incidence was found between the two groups. These findings suggest that an increase in AUC_0–24_ may not be directly associated with 90-day mortality or AKI risk. However, based on PK/PD principles, achieving an appropriate AUC_0–24_ is expected to enhance efficacy. Previous studies have linked adequate AUC_0–24_ to improved clinical outcomes of *Staphylococcus aureus* infections^10^ and reduced AKI incidence.^22^ In this study, AUC increased significantly without a rise in AKI incidence, indicating that the introduced support system may enhance efficacy while maintaining safety. Further large-scale studies with sufficient statistical power and subgroup analyses are warranted to clarify the clinical relevance of the observed AUC increase.

A limitation of this study is its single-center design. Future research should incorporate data from multiple institutions as this approach would enhance the generalizability and reliability of the findings.

In conclusion, the pharmacist-led support system increased the loading dose rate without negatively impacting AKI incidence or mortality, maintaining a balance between efficacy and safety. The improvement was immediate and sustained over the 122-week postintervention period. This system might serve as a practical model for broader administration in other healthcare settings.

## Data Availability

Data are available upon request and subject to privacy, ethics, and other restrictions.
